# Cutting-Edge Techniques and Drugs for the Treatment of Pulmonary Embolism: Current Knowledge and Future Perspectives

**DOI:** 10.3390/jcm13071952

**Published:** 2024-03-28

**Authors:** Lorenzo Falsetti, Emanuele Guerrieri, Vincenzo Zaccone, Giovanna Viticchi, Silvia Santini, Laura Giovenali, Graziana Lagonigro, Stella Carletti, Linda Elena Gialluca Palma, Nicola Tarquinio, Gianluca Moroncini

**Affiliations:** 1 Clinica Medica, Dipartimento di Scienze Cliniche e Molecolari, Università Politecnica delle Marche, 60126 Ancona, Italy; l.falsetti@staff.univpm.it (L.F.);; 2 Emergency Medicine Residency Program, Università Politecnica delle Marche, 60126 Ancona, Italy; e.guerrieri93@gmail.com (E.G.);; 3 Internal and Subintensive Medicine, Azienda Ospedaliero-Universitaria delle Marche, 60126 Ancona, Italy; 4 Clinica di Neurologia, Dipartimento Scienze Cliniche e Molecolare, Università Politecnica delle Marche, 60126 Ancona, Italy; 5 Internal Medicine Residency Program, Università Politecnica delle Marche, 60126 Ancona, Italy; 6 Internal Medicine Department, INRCA-IRCCS Osimo-Ancona, 60027 Ancona, Italy

**Keywords:** pulmonary embolism, submassive pulmonary embolism, catheter-directed thrombolysis

## Abstract

Pulmonary embolism (PE) is a potentially life-threatening condition requiring prompt diagnosis and treatment. Recent advances have led to the development of newer techniques and drugs aimed at improving PE management, reducing its associated morbidity and mortality and the complications related to anticoagulation. This review provides an overview of the current knowledge and future perspectives on PE treatment. Anticoagulation represents the first-line treatment of hemodynamically stable PE, direct oral anticoagulants being a safe and effective alternative to traditional anticoagulation: these drugs have a rapid onset of action, predictable pharmacokinetics, and low bleeding risk. Systemic fibrinolysis is suggested in patients with cardiac arrest, refractory hypotension, or shock due to PE. With this narrative review, we aim to assess the state of the art of newer techniques and drugs that could radically improve PE management in the near future: (i) mechanical thrombectomy and pulmonary embolectomy are promising techniques reserved to patients with massive PE and contraindications or failure to systemic thrombolysis; (ii) catheter-directed thrombolysis is a minimally invasive approach that can be suggested for the treatment of massive or submassive PE, but the lack of large, randomized controlled trials represents a limitation to widespread use; (iii) novel pharmacological approaches, by agents inhibiting thrombin-activatable fibrinolysis inhibitor, factor Xia, and the complement cascade, are currently under investigation to improve PE-related outcomes in specific settings.

## 1. Introduction

Pulmonary embolism (PE) is identified by the acute embolic obstruction of one or more pulmonary arteries with a very wide spectrum of clinical manifestations ranging from asymptomatic forms to severely impaired blood flow to the lungs and potentially life-threatening complications such as right ventricular (RV) dysfunction with shock and cardiac arrest. PE is often associated with deep vein thrombosis (DVT), an association defined as venous thromboembolism (VTE). It is estimated that PE affects up to 1 in 1000 individuals per year, with a mortality rate of approximately 10% in the first hour after onset of symptoms and up to 30% within the first month if left untreated [1,2].

PE diagnosis and management have undergone significant changes in recent years, and the development of diagnostic pathways and newer drugs have already improved patients’ outcomes [2,3]. Nevertheless, in-hospital mortality due to PE is still high, exceeding acute myocardial infarction [4]; this might suggest many potential areas of intervention, especially among patients with hemodynamic impairment [5].

The clinical complexity of the patient with PE encompasses several dimensions. Firstly, the degree of hemodynamic compromise in patients at high risk requires specific techniques to improve their short-term survival. Secondly, pharmacologic management can become even more complicated when PE is secondary or associated with other acute illnesses such as trauma, acute ischemic stroke, acute kidney injury, or thrombotic microangiopathies, which could radically modify the bleeding risk and the anticoagulant management. Thirdly, ageing and comorbidities such as anemia, cancer, chronic kidney disease, and coronary artery disease, coupled with related drug treatments, can worsen PE prognosis and increase the bleeding risk during treatments [6]. It is worth underlining that several risk scores engineered and validated to predict bleeding during PE (such as, for example, VTE-BLEED, PE-SARD, BACS, and RIETE) consider mainly age and other common chronic diseases as risk factors, underlining the central role of comorbidity in PE management [7,8,9,10].

To cope with such a complexity, the physicians actively involved in PE management should be aware of the most important research directions in this field, which are as follows: (i) organizational, with the constitution of pulmonary embolism response teams (PERTs) to manage the most difficult cases, (ii) technological, with the implementation of newer endovascular techniques, (iii) pharmacological, with the development of newer drugs able to improve the outcomes in specific clinical settings.

In the acute phase, several studies have explored the role of mechanical (MT) and catheter-directed thrombolysis (CDT) in the context of massive or submassive PE, and several devices are currently under investigation [5]. Both MT and CDT seem effective in treating patients with intermediate- or high-risk PE. However, more information from larger trials is required to completely support their use. 

MT is currently reserved for patients with massive PE with hemodynamic impairment in whom thrombolysis failed, in pregnancy, or with other contraindications to systemic thrombolysis [2,11]. MT devices involve the physical removal of the thrombus, either via manual extraction, maceration, or rheolytic fragmentation, and are associated with a fast reduction in pulmonary pressures and improved hemodynamics [12]. CDT is a minimally invasive technique that involves the delivery of thrombolytic agents directly into the pulmonary arteries via catheterization, which can dissolve the clot, reduce bleeding risk, and improve hemodynamics in subjects with massive and submassive PE [2,13].

Anticoagulation remains the mainstay of PE treatment at any level of severity: the aim of this treatment is to prevent further clot formation and promote the natural dissolution of the thrombus. Heparins (both unfractionated and low-molecular-weight), vitamin K antagonists (VKAs), and direct oral anticoagulants (DOACs) have been extensively studied, and their role has been clarified in several clinical settings [2]. The introduction of DOACs radically changed PE treatment, offering a safe and effective alternative to traditional anticoagulation agents such as heparin and VKA. DOACs have a rapid onset of action, predictable pharmacokinetics, and a lower risk of major bleeding. They have been shown to be non-inferior or superior to traditional anticoagulation therapy in large randomized controlled trials (RCTs) and are now recommended as first-line therapy for most patients with acute PE [2,13].

Fibrinolysis is currently suggested in high-risk PE, while its use in intermediate-risk is still object of debate. Recombinant tissue plasminogen activator (rtPA) is the most commonly studied and used drug both in systemic and in local fibrinolysis. Among fibrinolytics, tenecteplase has been recently evaluated in PE in several small studies and in a large RCT, the PEITHO trial [14]. Guidelines do not recommend this thrombolytic over the others in high-risk PE, and tenecteplase is not labeled for PE use [2,15]. In intermediate-risk PE, tenecteplase has been shown to be effective at the cost of a high bleeding risk in the PEITHO trial, with a low risk/benefit ratio [14].

Currently, the most debated topics in VTE management are represented by (i) the optimal treatment of patients with high- or intermediate–high-risk PE, (ii) PE management in subjects at a high risk of bleeding, (iii) the assessment and management of the bleeding risk in certain populations, and (iv) the management of VTE in specific, complement-mediated diseases. With this narrative review, we aim to explore current and investigational drugs and techniques that could improve PE management in the next few years.

## 2. Materials and Methods

The team of reviewers, after assessing the current literature and guidelines, selected the most evolving domains in PE treatment with three internal Delphi rounds. Upon selection of the items in an open questionnaire, a consensus was reached after three rounds, selecting the top five most-voted topics. Subsequently, pairs of reviewers were assigned to each item, focusing on an independent review of published material related to the assigned topic. Each group was allowed to select guidelines, original articles, randomized controlled trials (RCTs), meta-analyses, and high-quality reviews published in PubMed/EMBASE, Google Scholar, and Web of Science in the time frame between 2018 and 2023. Older but highly cited material was also allowed after consultation with the other reviewers.

## 3. Results

The board of reviewers selected the medical and surgical techniques to develop in this narrative review as follows: (i) mechanical thrombectomy, (ii) catheter-directed thrombolysis and ultrasound-facilitated catheter-directed thrombolysis, (iii) factor XI and XII inhibition, (iv) TAFIa inhibition, and (v) complement-modulating drugs. 

## 4. Discussion

### 4.1. Mechanical Thrombectomy

Massive PE is a life-threatening condition that requires prompt diagnosis and treatment. Although thrombolytic therapy is the standard treatment for massive PE, it is associated with a high risk of major bleeding, particularly in subjects with pre-existing contraindications to anticoagulation. MT is a technique that involves the use of catheters or devices to physically remove the clot; this method is emerging as a potential alternative to thrombolytic therapy for patients with high-risk PE or in specific groups of intermediate–high-risk PE. 

All the current guidelines recommend stratifying PE according to the risk of in-hospital death. This stratification is slightly different between the European and American guidelines, as shown in Table 1. However, the first-line treatment for all the classes of risk in all guidelines is represented by parenteral anticoagulation, and oral anticoagulation can be considered as an early treatment for subjects with a low or intermediate–low risk. The short-term efficacy of anticoagulation progressively decreases in patients with intermediate–high and high risk, mainly due to the time needed by these drugs to stabilize the thrombus and facilitate its dissolution. 

In patients with intermediate–high and high-risk PE, the most important aspect is the presence of various degrees of hemodynamic impairment. In this setting, the primary aim of the treatment is to reduce short-term mortality by rapidly reducing the clot burden and improving hemodynamic stability. While in high-risk PE, aggressive treatment with fibrinolytics has been shown to be beneficial; in intermediate–high-risk PE, trials did not show any benefit of early fibrinolysis, which was associated with an increased risk of life-threatening bleeding [16].

MT is an endovascular treatment characterized by techniques that differ according to the adopted device. This group of procedures takes advantage of guidewires commonly introduced into the femoral vein to reach the pulmonary veins. Once the clot is identified with a locally injected contrast medium, it is at least partially removed with a wire-guided aspiration system. This therapeutic strategy has shown a fast effect in improving hemodynamics and reducing right ventricle dysfunction with a low bleeding risk when compared to systemic thrombolysis [17,18]. 

The generic MT term comprises the following different catheters and techniques: (i) catheter-based thrombus maceration systems, (ii) rheolytic thrombectomy with AngioJet (Boston Scientific Corporation, Marlborough, MA, USA) catheters, (iii) large- and small-bore embolectomy with FlowTriever (Inari Medical Inc., Irvine, CA, USA) and Indigo (Penumbra Inc., Alameda, CA, USA) thrombectomy systems, and (iv) mechanical thrombectomy with Aspire Max (Control Medical Technology LLC, Park City, UT, USA) systems. The aim of all the above-mentioned devices is to remove the clot either by thrombus aspiration, with the addition of manual or mechanical pump, or by thrombus fragmentation. Specific devices can improve further clot reduction with the addition of low-dose thrombolytic agents [16].

MT devices such as FlowTriever have been shown to be effective in the reduction in the RV/LV ratio with a low rate of composite major adverse events and a low rate of readmissions within 30 days [17,18]. Furthermore, this strategy was able to significantly reduce the pulmonary pressures at baseline [17,18]. The FLASH study also underlined MT efficacy and effectiveness in patients with high-risk PE. Similarly, in the EXTRACT-PE trial, the primary efficacy endpoint (RV/LV ratio), the safety endpoint (rate of major bleeding), and the secondary endpoint (reduction in pulmonary hypertension from baseline to 48 h after the procedure) was confirmed, with the trial also adopting the Indigo aspiration device [19]. The effectiveness of the Aspire Max device was confirmed in a study that showed a rapid reversal of RV dysfunction in subjects with high- and intermediate–high-risk PE and a reduction in the long-term sequelae of persistent RV failure and pulmonary hypertension [20].

MT can be considered in cases of (i) hemodynamic instability, (ii) hemodynamic instability with contraindications to systemic and local thrombolysis, (iii) failure of thrombolysis, (iv) high thrombus burden, and (v) intermediate–high risk PE already treated with oral anticoagulants with hemodynamic deterioration or without improvement [2]. In high-risk PE, the 2019 ESC guidelines suggest surgical embolectomy with a higher class of recommendation (IC) than MT (IIaC), while the 2011 AHA guidelines suggest either surgical or mechanical techniques with the same class (IIaC) [2,15]. In patients with intermediate-risk PE, both the 2011 AHA guidelines and 2019 ESC guidelines suggest considering either surgical or mechanical embolectomy at the same level of evidence (IIaC in the ESC guidelines, IIbC in AHA guidelines) [2,15]. 

Subjects with a marked hemodynamic compromise, such as hypotension or shock, may not be able to tolerate a delay in symptomatic relief associated with pharmacological thrombolysis but may benefit from MT as a rapid and effective treatment option. In patients with evidence of hemodynamic compromise, these techniques may be associated and used in conjunction with drugs or other invasive hemodynamic support devices such as veno-arterial extracorporeal membrane oxygenation (VA-ECMO) or isolated percutaneous RV support during the procedure and until the hemodynamic stability can be warranted [16]. ESC guidelines suggest the consideration of use of fluids, vasopressors such as norepinephrine and inotropes such as dobutamine or milrinone, to achieve an hemodynamic improvement in the acute phase, with a low level of evidence (IIaC) [2]. More advanced circulatory supports can be useful for maintaining adequate circulation in the initial stabilization phase while planning and executing the intervention to remove the clot and during the recovery but are useless without an effective PE treatment [21]. These include VA-ECMO and mechanical circulation supports, such as the Impella RP device. The role of VA-ECMO in PE has been studied in several nonrandomized, single-center case series and observational studies and is currently suggested in association with surgical embolectomy by the ESC guidelines, with a very low level of evidence (IIbC). VA-ECMO diverts the venous flow outside the circulation, reduces the right ventricle dilatation, and improves systemic perfusion, thus stabilizing the subject [22]. Impella RP (Abiomed Inc., Danvers, MA, USA) has been successfully used as a mechanical support device for high-risk PE in case reports; however, a recent meta-analysis of the literature was not able to generalize its role in this setting, and ESC guidelines do not give a level of evidence for this treatment, suggesting its use only in selected cases [2,23]. Small studies suggest a potential role of both VA-ECMO and Impella RP in supporting hemodynamics in high-risk PE during MT and other endovascular procedures [24,25]. 

The absence of a proximal, reachable embolus and a previous reaction to contrast agent that cannot be adequately pretreated usually represent the absolute contraindications to MT. According to the FLASH registry, key clinical exclusion criteria for MT were also a low life expectancy and contraindications to systemic anticoagulation [26]. The other MT contraindications can be derived from the recently published trials that variably excluded patients with (i) contraindications to systemic anticoagulation (particularly with heparin), (ii) pulmonary hypertension with a peak pulmonary pressure >70 mmHg assessed with right heart catheterization, (iii) fraction of inspired oxygen >40% to maintain a saturation >90%, (iv) recent major trauma within the last 14 days, (v) recent cardiovascular or pulmonary surgery within the last seven days, (vi) active cancer, (vii) history of chronic pulmonary hypertension, (viii) low life expectancy (usually <30 days), (ix) known bleeding diathesis or coagulation disorders, and (x) low platelet count [18,19,26,27]. 

Although acute ischemic stroke (AIS) is infrequently associated with pulmonary embolism (PE), MT should be considered as a treatment option for patients with AIS and intermediate- or high-risk PE, despite the lack of guidelines and limited data on topic [28].

MT techniques should be considered for patients who have absolute or relative contraindications to local and systemic fibrinolysis. However, in this setting, some technical aspects must be taken into account. First, blood loss from aspiration-based MT can limit effective thrombus removal in complex procedures: studies have shown that incorporating blood return systems significantly reduces blood loss during procedures without impacting blood pressure or cardiac index. Interestingly, these findings suggest that the clot burden of acute PE is more closely associated with hemodynamic stability than blood loss resulting from the procedure [29]. Second, it is crucial to emphasize that the mortality rates and occurrences of major bleeding are comparable between catheter-directed thrombolysis (CDT) and mechanical thrombectomy (MT). This observation suggests that MT may not provide a significant advantage in reducing severe bleeding risk in comparison to CDT, except in cases where thrombolytics are entirely contraindicated [11,30,31]. 

MT outcomes in intermediate–high and high-risk PE are generally favorable, with high rates of success in clot removal and improvement in hemodynamic stability. MT is associated with a low risk of complications, including bleeding (both systemic and in the access site), perforation of the pulmonary artery, distal clot displacement with worsening of hemodynamic stability and RV dysfunction, and systemic embolization of clot fragments [17,18]. 

The extreme complexity of the management of patients with high-risk or intermediate–high-risk PE often requires the organization of rapid response teams able to perform a multidisciplinary approach: PERTs are heterogeneous teams of cardiologists, pneumologists, hematologists, interventional radiologists, emergency medicine doctors, intensive care specialists, vascular and cardiac surgeons that are called to formulate a treatment plan in difficult-to-treat cases [2]. The implementation of PERTs has been associated with an increased use of advanced treatments such as MT, reduced inferior vena cava filter positioning, and a trend towards reduced mortality [32].

The long-term outcomes of MT, including mortality and the risk of recurrent PE, are still not well established. The principal limitation of MT is the absence of randomized controlled studies with an adequate sample size. Most of the performed RCTs limited their observations to the short-term outcomes, and were able to demonstrate strong evidence of this technique in improving hemodynamics after the procedure, as in the EXTRACT-PE [19].

Only some currently adopted MT systems are both FDA-approved and CE-certificated for PE. Moreover, some systems are being used off-label in this context, being indicated for other purposes or on other sites. Currently, two systems have obtained both FDA approval and a CE mark (Penumbra Indigo Aspiration and Inari Medical FlowTriever). Two other devices have CE approval for PE treatment (Aspirex, Straub Medical AG, Wangs, Switzerland; Angiojet, Boston Scientific Corporation, Marlborough, MA, USA) [33,34]. Other systems are approved for the non-surgical removal of emboli and thrombi from blood vessels but are not specifically indicated for PE. Among these, it is worth mentioning the Angiovac system (Angiodynamics Inc., Latham, NY, USA), which is currently indicated to remove vascular material from the superior and inferior vena cava and the right atrium, but it has also been used off-label for MT in PE [34,35].

Several manufacturers are actively engaged in engineering more efficient MT devices, improving existing systems and developing novel techniques. Rheolytic MT with Angiojet obtained a black-box warning from the FDA for PE use after reports of severe adverse events (asystole and hemodynamic decompensation). However, technical developments are improving this technique and its outcomes [34]. The Alphavac system (Angiodynamics Inc., Latham, NY, USA) is currently approved for thrombus removal from peripheral veins, and is being assessed for PE clot removal in a clinical study [36]. The Akura MT platform (Akura Medical Inc., Los Gatos, CA, USA) has been proven effective in a first-in-human trial [37]. The Magneto eTrieve (Magneto Thrombectomy Solutions, Yehuda, Israel), which combines large bore aspiration with electro-mechanical thrombus extraction, has proven to be effective in a first-in-human study [38]. The Bashir pharmacomechanical approach (Thrombolex Inc., New Britain, PA, USA) resulted in being effective in the RESCUE trial, and recently received FDA clearance for the treatment of acute PE [39]. 

The literature still requires a better definition of patients’ selection criteria and a more extensive evaluation of long-term outcomes of MT-treated subjects, as discussed more extensively in a recent, focused review [33]. The use of MT in combination with thrombolysis or other anticoagulant treatments needs larger studies. The CANARY study, specifically designed to address this topic, was interrupted due to the surge of the 2020 COVID-19 pandemic [40]. Despite these limitations, MT represents an effective option for patients with massive PE at high risk of complications or contraindications of thrombolytic therapy. The procedure is associated with a high rate of clot removal and an improvement in hemodynamic stability, although it is also associated with a risk of complications [17,18,19].

### 4.2. Catheter-Directed Thrombolysis

Catheter-directed thrombolysis (CDT) is a minimally invasive technique that involves the insertion of a catheter into the pulmonary artery to deliver a thrombolytic agent, such as rtPA, directly to the site of the blood clot, aiming to quickly restore the lung perfusion. By delivering the thrombolytic agent locally, CDT can achieve higher concentrations of the drug at the clot site, minimizing systemic exposure and reducing the bleeding risk [41].

European (ESC) and American (AHA) guidelines suggest considering CDT use in patients with high- or intermediate–high risk PE associated with hemodynamic and respiratory deterioration despite anticoagulation [2,15], but also in subjects with high-risk PE with contraindications to systemic rtPA or in whom systemic fibrinolysis has failed [2,16]. In this context, the most adopted ranking system for patient’s selection is represented by the 2019 ESC classification [2]. Although all guidelines agree in categorizing massive or high-risk PE as the forms with clinically significant hemodynamic instability characterized by persistent hypotension, cardiac arrest, or obstructive shock, there is a lack of consensus regarding the definition of the intermediate or submassive forms, as shown in Table 1.

Several studies have evaluated CDT efficacy and safety in patients with intermediate–high or high-risk PE with different interventional devices, as synthesized in Table 2, panel A.

#### 4.2.1. Catheter Directed Thrombolysis (CDT)

- In a pilot RCT, CDT with 20 mg rtPA was compared with anticoagulation in 23 patients with intermediate–high-risk PE [42]. The primary efficacy endpoint, measured at 48 h after randomization, was defined as a ≥25% reduction in the RV/LV ratio, a reduction in echocardiographic-estimated pulmonary pressure (sPAP) by 30% from baseline, or the achievement of normal systolic pulmonary pressure, or a ≥30% reduction in the Qanadli score. Safety was assessed by evaluating the absence of intracranial or life-threatening bleeding [33]. The limitations of the study were the reduced sample size and the lack of clinical endpoints. However, CDT appeared to be safe and effective in this setting [42].
- The CANARY trial aimed to perform a larger comparison between CDT and anticoagulation in patients with intermediate–high-risk PE. The protocol for the CDT group involved the administration of 12 mg rtPA for unilateral PE or 24 mg for bilateral PE over 24 h. On the other hand, the anticoagulation group received enoxaparin at standard doses. However, due to the COVID-19 pandemic, the RCT was prematurely interrupted. Despite this, the study was not able to detect a statistically significant difference between anticoagulation monotherapy and CDT regarding the primary endpoint of the proportion of patients with a 3-month RV/LV ratio of greater than 0.9. Nevertheless, the RCT showed a low risk of major bleeding in the CDT arm and an improvement in the 3-month RV echocardiographic recovery [40].
- In a meta-analysis comprising six studies, the efficacy of CDT versus standard anticoagulation was compared with 30-day, 90-day, and one-year mortality rates, as well as the occurrence of major bleeding events. The results of the study highlighted the benefit of CDT over standard anticoagulation at 30-day and one-year mortality, while the 90-day mortality rate remained similar between the two groups. Notably, the study observed that the rate of major bleeding events was comparable between the two groups [43].

#### 4.2.2. Ultrasound-Assisted Catheter-Directed Thrombolysis (US-CDT)

In vitro studies have suggested that ultrasonic waves have the potential to enhance thrombolytic penetration and increase thrombus dissolution during fibrinolysis. Based on these observations, manufacturers have developed the EkoSonic Endovascular System (EKOS) with the objective of delivering both acoustic energy and lytic agents directly to the clot to facilitate its dissolution [44,45]. US-CDT could favor the separation of fibrin strands, allowing the improvement of the dose-effect relationship of fibrinolytic agents and maximizing the effectiveness of thrombolytic therapy [33]. The alleged clinical benefit of this method is that comparable thrombus clearance may be attained using lower doses of lytic agents and/or shorter duration of therapy, potentially leading to a reduction in complication rates and hospital stay.

- The ULTIMA trial compared US-CDT plus unfractionated heparin (UFH) anticoagulation to UFH in 59 patients with an intermediate–high risk PE and RV/LV ratio ≥1. The authors observed in the US-CDT group a significant reduction in RV strain and pulmonary artery pressure, a lower bleeding risk than systemic thrombolysis [46], a lower rate of recurrent PE in the CDT group and a non-significant trend towards lower mortality.
- In the PERFECT registry, 101 PE patients were prospectively enrolled and treated with catheter-directed mechanical or pharmacomechanical thrombectomy and/or CDT using rtPA or urokinase. In the study, 85.7% of patients with massive PE and 97.3% of patients with submassive PE achieved clinical success, defined as hemodynamic improvement, reduced pulmonary pressure, reduction in right ventricular dilation, and survival to hospital discharge. However, no significant differences in sPAP reduction were observed between the CDT and US-CDT groups [47].
- The SUNSET sPE RCT aimed to evaluate the efficacy of US-CDT in reducing the thrombotic burden in patients with intermediate–high risk PE, as compared to traditional CDT. The study did not show a significant difference in the reduction in the pulmonary arterial thrombus, measured by the change in the Miller scoring system, between groups. However, both methods were found to produce a significant improvement in RV function, with a superior RV/LV ratio reduction observed in the US-CDT group. It remains unclear whether US-CDT use produces a better lytic effect that provides a clinical advantage significant enough to justify the higher cost of the catheter as compared to the traditional one [48].

**Table 2:** Relevant studies on CDT and US-CDT.

Study	Device	FDA/CE [33,34]	Cohort	Comparison	Analyzed Outcomes
Panel A: Published studies					
Kroupa [42]	Cragg-McNamara	A_GEN_/M_GEN_	IR-PE	CDT (rtPA) + UFH versus UFH or LMWH	RV/LV ratio, sPAP, Qanadli score, and safety (intracranial or life-threatening bleeding)
CANARY [40]	Cragg-McNamara	A_GEN_/M_GEN_	IR-PE	CDT (rtPA) + UFH versus LMWH	RV/LV ratio, RV recovery
ULTIMA [46]	EKOS	A_PE_/M_PE_	IR-PE	US-CDT (rtPA) + UFH versus UFH	RV/LV ratio
SUNSET [48]	Cragg-McNamara Uni-Fuse EKOS	A_GEN_/M_GEN_ A_PE_/M_GEN_ A_PE_/M_PE_	IR-PE	CDT (rtPA) versus US-CDT (rtPA)	RV/LV ratio, Miller score, intensive care unit stay, in-hospital stay, bleeding, and adverse events up to 90 d
Panel B: Ongoing studies					
BETULA [49]	Uni-Fuse	A_PE_/M_GEN_	IR-PE	CDT (rtPA) versus UFH	RV/LV ratio, lung perfusion, LOS, 30 d mortality, recurrent PE, LOS, reduction in embolic extension
PE-TRACT [50]	MT or CDT	--	IR-PE	MT or CDT versus AC	PVO2, NYHA class, incidence of major bleeding within 7th day
HI-PEITHO [51]	EKOS	A_PE_/M_PE_	IR-PE	US-CDT (rtPA) + UFH versus LMWH o UFH	PE-related death, PE decompensation, PE recurrence
STRATIFY [52]	US-CDT	--	IR-PE	US-CDT (rtPA) + UFH or LMWH versus LDT + UFH or LMWH versus UFH or LMWH	Miller Score

Legend: 30 d: 30-day; 90 d: 90-day; A_GEN_: FDA-approved for generic endovascular use; A_PE_: FDA-approved for PE; Cragg-McNamara: MedTronic, Inc., Minneapolis, MN, USA; AC: anticoagulant; CDT: catheter-directed thrombolysis; IR-PE: intermediate–high-risk pulmonary embolism; EKOS: EkoSonic Endovascular System, Boston Scientific Corporation, Marlborough, MA, USA; LDT: low-dose thrombolytics; LOS: length of hospital stay; LV: left ventricular; M_GEN_: CE mark for generic endovascular use; M_PE_: CE mark for PE; NYHA: New York Heart Association; RV: right ventricular; sPAP: systolic pulmonary artery pressure; PE: pulmonary embolism; LMWH: low molecular weight heparin; UFH: unfractionated heparin; Uni-Fuse: Angiodynamics Inc., Latham, NY, USA; US-CDT: ultrasound-assisted catheter-directed thrombolysis; PVO2: peak oxygen consumption; rtPA: recombinant tissue plasminogen activator.

- In the SEATTLE II trial, 150 patients with submassive and massive PE were enrolled to study the efficacy and safety of US-CDT plus standard anticoagulation. The results showed that the US-CDT procedure significantly reduced the RV/LV ratio, the number of patients with right ventricular disease, and the sPAP after 48 h, in association with a low rate of recurrent PE and chronic thromboembolic pulmonary hypertension at 6 months. However, 10% of the population showed major bleeding events [13,53].
- The OPTALYSE RCT was conducted to assess the efficacy of US-CDT in patients with intermediate-risk PE. The study focused on identifying the optimal rtPA dose and delivery duration for US-CDT. The findings revealed that using a lower rtPA dose and a shorter duration of administration in US-CDT led to improved right ventricular function and a reduction in clot burden when compared to baseline. Although the rate of major bleeding was low, one intracranial hemorrhage event attributable to US-CDT treatment and four cases of major bleeding were described [54].

Therefore, the current literature supports the concept that CDT may improve overall outcomes for patients presenting with PE and an indication for aggressive management. The conclusions of a meta-analysis of eight observational studies considering more than 10,000 subjects with high-risk or intermediate–high-risk PE are that (i) CDT appears to be associated with improved in-hospital mortality compared to systemic fibrinolysis for patients with massive or submassive PE, (ii) rates of major bleeding do not appear to differ by thrombolysis delivery strategy, and (iii) intracranial hemorrhage rates appear to be lower for those receiving CDT. While in-hospital survival appears to be improved with CDT, this difference is not explained by a global reduction in major bleeding events. Furthermore, the small but significant reduction in intracranial hemorrhage seems not to account for the observed survival differences [55]. 

Despite these observations, real-world data suggest an underutilization of reperfusion treatments [56], and this point should be improved, also with the local implementation of PERTs. In fact, nowadays, CDT is feasible even in subsets of patients at very high bleeding risk, such as, for example, in subjects treated with double antiplatelet treatment or with chronic liver disease, following specific guidelines [57].

CDT appears to be a promising therapeutic intervention for intermediate–high-risk PE: catheter delivery may offer a more complete thrombolysis, and the modality of infusion is markedly different from systemic thrombolysis. CDT physically steers drug delivery to the thrombus for thorough infiltration, making it superior to systemic thrombolysis for the treatment of intermediate–high risk PE [55]. However, the procedure carries significant risks, including bleeding, and still lacks standardization in dosing and duration of thrombolytic therapy. It requires specialized equipment and expertise that can be unavailable in smaller hospitals and may be time-consuming and costly. Careful evaluation of patient suitability is necessary before proceeding with the procedure: in particular, patients with a history of bleeding disorders, recent surgery, or trauma should be carefully evaluated before CDT.

The existing data regarding CDT are not sufficient to establish these options as first-line treatments for patients with intermediate-risk PE. An important gap in the evidence is the lack of demonstration of the clinical benefits in terms of positive impact on prognosis and quality of life using a valid, composite clinical outcome [58]. For this reason, the decision to use CDT should be carefully evaluated on a case-by-case basis, taking into consideration the patient’s clinical status, the size and location of the clot, and the potential risks and benefits of the procedure.

Several aspects of CDT are under evaluation with different RCTs that aim to compare the efficacy and safety of CDT techniques with the current standard of care, anticoagulation therapy, as shown in Table 2, panel B. HI-PEITHO is a multinational multicenter randomized controlled trial aiming to assess whether US-CDT in combination with anticoagulation therapy reduces the composite outcome of mortality, cardiac or respiratory instability, or nonfatal symptomatic and objectively confirmed PE recurrence compared to anticoagulation therapy alone, within 7 days of randomization. This is the only ongoing trial directly comparing US-CDT with systemic anticoagulation with heparin. If the treatment arm of HI-PEITHO is confirmed to be superior to the control arm, US-CDT will have provided, for the first time, solid evidence to establish it as a first-line treatment in this category of subjects [51].

### 4.3. Inhibition of Factor XI and Factor XII

Currently, MT and CDT are considered the most advanced techniques to treat high-risk and intermediate-risk PE. However, researchers are exploring newer categories of anticoagulants to improve the management of low-risk or intermediate–low-risk PE. The inhibition of factor XI (FXI) and factor XII (FXII) are currently being considered a significant breakthrough in the prevention and treatment of both PE and DVT. According to recent clinical trials, it has been suggested that FXI inhibitors could demonstrate superior efficacy and safety in comparison to conventional anticoagulants, including DOACs. Thus, this heterogeneous category of drugs may have a potential application in specific categories of subjects, such as patients with cancer. The treatment of cancer-associated PE still represents an unmet clinical need since the actual treatments, represented by low-molecular-weight heparins (LMWH) and DOACs, are burdened by a significant drop-out rate and a consistent number of major bleedings, respectively [59]. 

**Figure 1:**
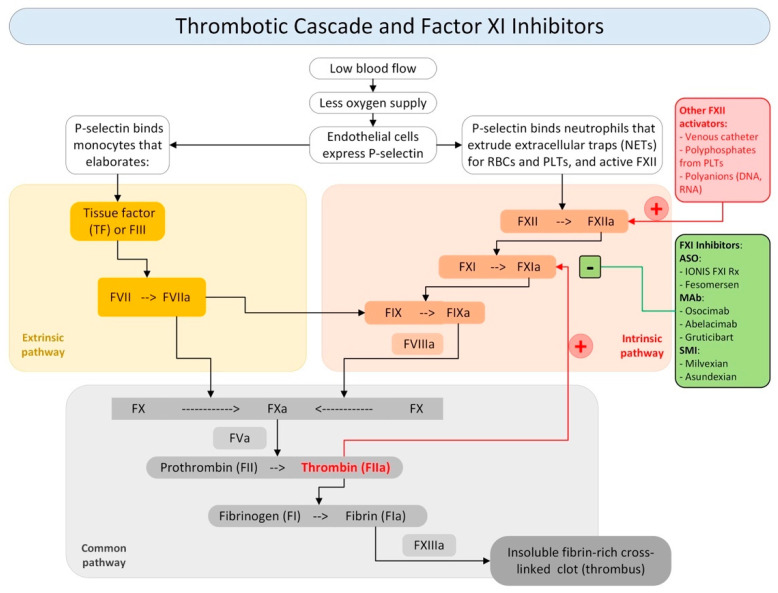
Thrombotic cascade and role of Factor XI inhibitor. Legend: NETs: (+) activators; (−) inhibitors; neutrophils extracellular traps; RBCs: red blood cells; PLTs: platelets; F: factor; TF: tissue factor; ASO: antisense oligonucleotides; MAb: monoclonal antibodies; SMI: small molecule inhibitors.

VTE occurs from the activation of two pathways of the coagulation cascade, namely the extrinsic and intrinsic pathways, which leads to the activation of the common pathway responsible for thrombus formation [60,61,62,63,64,65]. The inhibition of FXI and FXII would not only block the normal action of the two factors within the coagulation cascade but would also prevent the self-feeding phenomena of the intrinsic pathway, thus reducing the probability of thrombus formation or propagation, as shown in Figure 1.

The use of FXI and FXII inhibitors is made even more attractive by their ability not to significantly affect hemostasis. Studies show that a FXII deficiency does not lead to a bleeding tendency. In contrast, people with FXI deficiency may tend to bleed, but the likelihood of bleeding is still very low [66]. Although this evidence suggests a slight increase in safety in the use of an FXII inhibitor compared to the use of an FXI inhibitor, recent clinical trials have placed a greater emphasis on FXI inhibition for several favorable factors. Previous works have shown an association between high FXI concentrations and VTE, but not between FXII levels and thrombosis [67,68,69,70].

Some authors have observed that FXI deficiency was associated with a lower VTE risk, while high FXI levels increased the risk. On the other hand, data regarding the impact of FXII levels on VTE are less straightforward [71,72]. In addition, FXI’s ability to be activated not only by FXII but also by thrombin may make FXII inhibitors less effective. Considering the narrow connection between coagulation, complement, and inflammation pathways, FXI inhibition could be considered in future trials as a potential approach for complement-mediated thromboses, such as antiphospholipid antibody syndrome (APS), and proinflammatory processes, such as cancer-associated thrombosis.

Several types of FXI inhibitors have been proposed and each pharmacological category has its own benefits and limitations. We can distinguish three main classes of FXI inhibitors: (i) antisense oligonucleotides; (ii) monoclonal antibodies; and (iii) small molecule inhibitors. The principal molecules acting on this pathway are shown in Figure 1.

Drugs of the first category include Ionis FXI Rx and Fesomersen. These drugs act to reduce FXI hepatic synthesis by inducing the catalytic degradation of FXI mRNA. They are administered parenterally and have a slow onset and offset. The second category is represented by Osocimab, Abelacimab, and Gruticibart (Xisomab/AB023), and acts by suppressing FXIa generation or by inhibiting FXIa activity or with both mechanisms of action. They are administered parenterally and have a rapid onset and a slow offset. The third category are represented by Milvexian and Asundexian and acts by blocking the active site of FXIa or by inducing allosteric modulation. They are administered orally and have a rapid onset and relatively rapid offset [73,74,75,76,77]. 

For their modality of action, their presumed low risk of bleeding, and their long life, these drugs are currently under investigation in phase 3 trials for the treatment of cancer-associated PE. Currently, there is insufficient information available regarding the comparative efficacy and safety of FXI inhibitors in contrast to DOACs, particularly apixaban. However, the data obtained from phase 2 clinical trials suggest that FXI inhibitors may produce a better antithrombotic effect and a lower bleeding risk, as compared to DOACs [78]. Abelacimab is the first FXI inhibitor studied in a phase 3 RCT. The ASTER and MAGNOLIA trials are ongoing, aiming to assess the efficacy and safety of abelacimab compared to apixaban and dalteparin to treat cancer-associated PE in a subset of patients at high- or very-high risk of bleeding [79,80]. The interest in this molecule in the setting of cancer-associated PE should be related to its ease of use, as it requires only a single monthly administration; moreover, it is not dependent on hepatic and renal metabolism, it does not interact with other drugs, and it could be also be used in patients with severe kidney insufficiency [78]. These drugs are also under investigation for VTE prophylaxis in patients undergoing hip or knee replacement surgery: currently, phase 2 RCTs and exploratory meta-analyses have shown a 40–50% reduction in VTE in patients treated with FXI inhibitors compared with enoxaparin and a 59% reduction in bleeding [81,82]. 

### 4.4. Thrombin Activatable Fibrinolysis Inhibitor

The effectiveness of local and systemic fibrinolysis in patients with intermediate-risk PE remains uncertain due to the potential risk of bleeding: the current indication of systemic fibrinolysis in this subgroup is for subjects undergoing hemodynamic destabilization despite standard anticoagulation and interventional treatments should be considered in those who need a rescue treatment but have contraindications or failed to respond to systemic treatments [34,83,84]. In order to improve the outcomes in this area of therapeutic uncertainty without increasing the bleeding risk, several preclinical studies have focused on inhibiting antifibrinolytic molecules to enhance endogenous thrombolytic activity.

Thrombin activatable fibrinolysis inhibitor (TAFI) is classified as a circulating zinc-dependent metallocarboxypeptidase protein. TAFI production occurs through proteolytic cleavage by trypsin-like proteases, such as thrombin or plasmin [85]. During the coagulation cascade, TAFI becomes activated and plays a crucial role in the formation and stabilization of clots. TAFI undergoes activation by the thrombin–thrombomodulin complex following thrombin generation. Its activated form (TAFIa) is responsible for its antifibrinolytic properties, which are attributed to its carboxypeptidase activity [86]. TAFIa inhibits fibrinolysis by preventing plasminogen and tPA binding and reducing plasmin [87]. 

**Figure 2:**
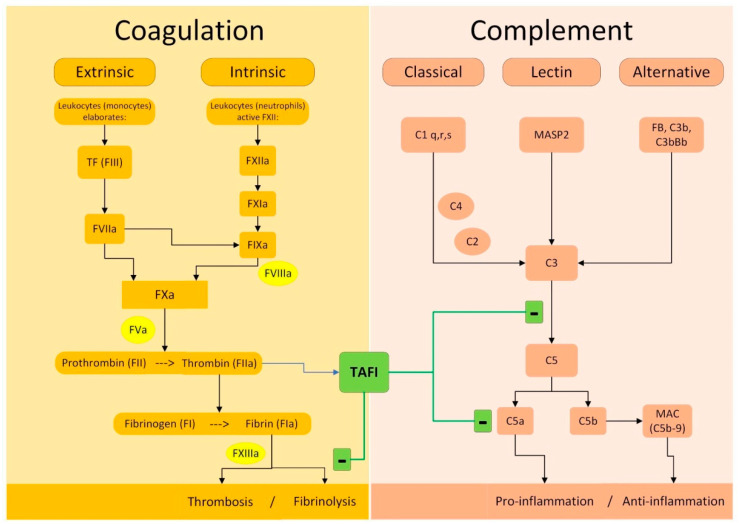
Molecular mechanisms of TAFI on coagulation and complement. Legend: (+) activation; (−) inhibition; TAFI is activated to TAFIa by thrombin and exerts its actions on both fibrinolytic and complement system.

TAFIa serves a dual purpose as both an antifibrinolytic protein and a critical mediator of anti-inflammatory processes, as illustrated in Figure 2. Given that TAFI is activated by thrombin and downregulates the fibrinolytic response, it represents a homeostatic link between inflammation, coagulation, and fibrinolysis, playing a crucial role in maintaining the delicate balance between clot formation and dissolution. This ability underscores the potential therapeutic implications of targeting TAFIa for managing the pro-thrombotic states observed in several inflammatory disorders [88,89]. Given its antifibrinolytic properties and its potential association with thrombotic events and cardiovascular diseases, TAFIa inhibition represents a promising target for pharmacological intervention in PE. Drugs under development entail exploring two primary strategies: TAFI activation inhibition and TAFIa inhibition. The three main categories of drugs acting on this pathway are represented by synthetic peptides, small-molecule inhibitors, and antibodies [90,91]. Most of the above-mentioned drugs are still under investigation, at a very early, preclinical stage.

The small-molecule inhibitor DS-1040 represents the most advanced clinical application of TAFIa inhibition to date. DS-1040, a low-molecular-weight imidazole derivative, can inhibit the enzymatic activity of TAFIa, leading to a potentiation of endogenous tissue plasminogen activator-triggered fibrinolysis. DS-1040 has been evaluated in humans in cohorts of healthy subjects [92], but also in acute ischemic stroke [93] and hemodynamically stable, intermediate-risk PE [94] in phase 1 RCTs. 

Despite an intriguing mechanism of action, however, DS-1040 use in PE did not reduce PE thrombus volume, and there was no difference in the change in RV/LV ratio when comparing results versus standard anticoagulation. The addition of DS-1040 to standard treatments for PE and acute ischemic stroke did not significantly increase the number of clinically significant bleedings, suggesting a good safety profile of this molecule. However, since the mechanisms of action of TAFIa inhibitors are slightly different between molecules and drug categories, further studies are required to assess whether TAFIa inhibition could be an interesting target to improve the management of intermediate-risk PE.

### 4.5. Inhibition of Complement Activation

PE may be accompanied by rare clinical conditions associated with complement dysregulation that could pose significant challenges in its management in clinical practice. These challenges may arise due to factors such as low platelet count, anemia, and multi-organ failure that limit the treatment options available and make it difficult to manage this condition effectively. 

**Figure 3:**
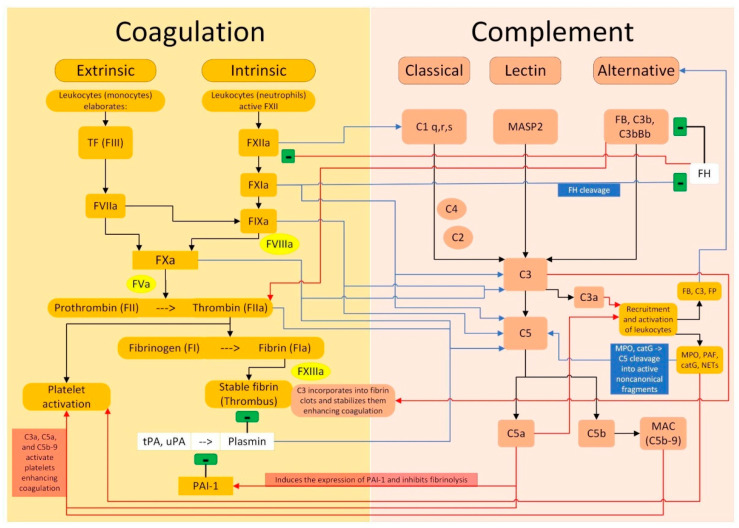
Crosstalk between the coagulation pathway and the complement pathway. Legend: (i) black arrows indicate an activation between the elements of the same pathway; black lines with the final “−” symbol indicate an inhibition between the elements of the same pathway, both for the coagulation and for the complement pathways; (ii) blue arrows indicate that an element of the coagulation pathway activates one or more elements of the complement pathway; the blue line with the final “−” symbol indicates that an element of the coagulation pathway inhibits an element of the complement pathway; (iii) the red arrows indicate that an element of the complement pathway activates one or more elements of the coagulation pathway; the red line with the final “−” symbol indicates that an element of the complement pathway inhibits an element of the coagulation pathway.

The complement is a complex system that plays a critical role in innate and adaptive immunity. The coagulation intersects the complement cascade at multiple points, as shown in Figure 3. When the downregulation mechanisms of the complement cascade are lost or become less efficient, an uncontrolled complement activation increases the risk of arterial and venous thrombosis. This phenomenon, known as immunothrombosis, presents clinically a wide range of manifestations, including DVT and PE, as well as thrombotic microangiopathy (TMA) and arterial thrombosis.

The association between complement dysregulation and activation of the coagulation pathway has been paradigmatically described in three rare and life-threatening diseases: paroxysmal nocturnal hemoglobinuria (PNH), autoimmune hemolytic anemia (AIHA), and atypical hemolytic uremic syndrome (aHUS). In these conditions, mortality and morbidity are mainly due to (i) large-vessel arterial and venous thrombosis, with a high PE prevalence, (ii) TMA with end-organ insufficiency, (iii) major bleeding due to a low platelet count. 

PNH is associated with the loss of two complement-inhibiting surface proteins (CD55 and CD59) expressed mainly on the surface of red blood cells and leading to a dysregulated complementary activation that results in hemolysis and thrombosis [95]: VTE events are common, affecting 29–44% of the subjects, and associated with an increased mortality. A total of 25% of the thromboses are arterial, with myocardial and cerebral arteries most commonly affected, while 75% of the cases are venous (hepatic, portal, cerebral, and lower limbs), with 14% of patients manifesting with PE [96]. AIHA is mainly characterized by hemolysis [97], but complement can be particularly activated in patients with cold agglutinin disease (CAD). In this subset, there is a markedly increased risk of arterial and venous thrombosis (11–27% of cases), especially in the presence of APS or splenectomy. A total of 75% of the thrombotic events are venous (mainly splanchnic and lower-limb veins), with 33% of PE, while 25% affect the arterial bed, with coronary or cerebral artery involvement [96]. aHUS is characterized by dysregulated complement activation, which primarily occurs on the endothelium and is often triggered by systemic events, manifesting mainly as a TMA, but can also affect arterial and venous system with acute thrombotic events. The exact PE prevalence in aHUS is not known due to the low prevalence of this disease. However, PE has been associated with a worse prognosis in this setting [98,99].

Other autoimmune disorders, such as APS and catastrophic antiphospholipid syndrome (CAPS), are associated with a substantial complement activation and a subsequently increased VTE incidence. APS is associated with both arterial and venous thrombotic events: DVT is observed in 38.9% of the cases, while PE reaches a prevalence of 14.1% [100]. Catastrophic antiphospholipid syndrome (CAPS), a rare and life-threatening variant of antiphospholipid syndrome (APS), is characterized by a sudden onset of symptoms, macrovascular and microvascular involvement which is often accompanied by arterial and venous thrombosis. PE has been reported in 26% of CAPS cases [101]. Dysregulation of complement activity has also been described in other autoimmune diseases, such as ANCA-associated vasculitides, and in non-autoimmune pathologies, such as severe COVID-19 and unprovoked VTE [102,103,104,105,106].

Modulation and inhibition of complement activity has been extensively studied in the setting of immunothrombosis and can be achieved with different pharmacological approaches: the most studied drug is eculizumab, a humanized recombinant monoclonal antibody binding C5 and preventing the initiation of the terminal cascade activation by inhibiting the C5a fragment release. Ravalizumab is a newer monoclonal antibody with a similar mechanism of action but with better pharmacokinetics that can be used as an alternative to eculizumab. 

PE is common in PNH, aHUS, and AIHA, especially during the acute flares that are associated to a more evident complement dysregulation [107]. VTE occurrence has been associated with increased mortality and morbidity in this setting, and the worse prognosis has been attributed to several factors, such as (i) anemia, (ii) severe thrombocytopenia, (iii) reduced response to anticoagulant therapy, (iii) markedly increased bleeding risk, and (iv) multi-organ failure. Moreover, the association between complement dysregulation syndromes and PE occurs often in already difficult-to-treat patients, such as in pregnant women and markedly thrombocytopenic subjects. In these difficult-to-treat contexts, PE should be managed in the context of PERTs, using a multidisciplinary approach considering endovascular techniques, such as MT and VA-ECMO in combination with anticoagulants, steroids, plasma exchange, and anticomplement treatments. The combination of these interventions is often necessary to improve hemodynamics and reduce complement activity to achieve better control of coagulation, immunothrombosis, and end-organ disease. The use of different techniques has been successfully described in PE cases of difficult management [108,109]. However, the role of MT and CDT in these cases, especially in the presence of severe thrombocytopenia, remains uncertain, and management strategies should be tailored to the patient’s clinical characteristics [110].

PNH has the strongest evidence of benefit from complement modulation: eculizumab and ravalizumab improve survival and disease control, are suggested as the emergency treatment in case of PE or DVT, and significantly reduce the rate of VTE events [111,112,113]. In this specific clinical context, life-threatening PE has been successfully treated in case-series with complement modulation, anticoagulation, and CDT. However, this strategy has been associated with an increased risk of manageable bleeding [114]. 

In acute symptomatic AIHA, the first-line treatment of cases presenting with DVT or PE should be represented by anticoagulants and steroids. However, especially in CAD, complement-modulating drugs should be considered as part of the initial treatment to reduce hemolysis and immunothrombosis [115]. In aHUS, emergency treatment with complement-modulating drugs is always necessary to control TMA and its associated complications (end-organ disease, arterial and venous thrombosis), raising platelet count, improving hemostasis, and reducing microvascular hemolysis [116,117]. Case reports suggest that a strategy considering endovascular techniques and complement-modulating drugs could also be effective in aHUS complicated by life-threatening PE [108].

APS and CAPS are immune-mediated disorders in which complement activation has been observed as part of the pathogenetic process, being associated with markedly raised VTE risk, reduced clinical response to anticoagulants, and TMA-like characteristics with thrombocytopenia. Some studies have explored the role of complement modulation with positive—albeit preliminary—results. The current treatment of symptomatic APS is based mainly on anticoagulants and antiplatelet drugs; however, treatment with eculizumab has been successfully added to a multidrug strategy in refractory forms [102,118,119]. A severe dysregulation of complement activity has been described in CAPS, and treatment with eculizumab has been associated with a reduced risk of death in the acute phase of disease and a reduced VTE recurrence in the follow-up [120]. Eculizumab seems more effective in subjects with CAPS showing thrombocytopenia with TMA and in those who are refractory to first-line treatments, represented by anticoagulation, corticosteroids, intravenous immunoglobulins, or plasma exchange [120,121]. 

## 5. Conclusions

PE treatments are rapidly evolving. MT and CDT are actual techniques that are already improving the management of high- and intermediate–high-risk PE. Newer pharmacological treatments, such as FXIa and TAFIa inhibitors, are currently under investigation to improve PE management in specific clinical settings, while complement-modulating drugs currently represent a therapeutic mainstay in immunothrombosis, in which PE represents a very difficult-to-treat complication. Such clinical, pharmacological, and technological complexity suggests that centers that aim to manage PE successfully should set up multidisciplinary groups, also known as pulmonary embolism response teams (PERTs), in order to improve the decision-making process and case management, especially in patients at a high- or intermediate–high risk.

## Figures and Tables

**Table 1:** Current PE classification according to hemodynamic status.

	Hemodynamic Alterations	Right Ventricle Dysfunction	Biomarkers (TnI or BNP)	PESIc > III or sPESI > 1
American Heart Association				
Low-risk	−	−	−	N/A
Submassive	−	+/−	+/−	N/A
Massive	+	N/A	N/A	N/A
European Society of Cardiology				
Low-risk	−	−	−	−
Intermediate-low risk	−	+/−	+/−	+
Intermediate-high risk	−	+	+	+
High-risk	+	N/A	N/A	N/A

Legend: BNP: (−) the item should not be present to define the hemodynamic category; (+/−) can be useful to refine the stratification; (+) the item is necessary to define hemodynamic category; serum brain-derived natriuretic peptide; N/A: not adopted by the guidelines or not necessary; PESI: pulmonary embolism severity index; PESIc: PESI category; sPESI: simplified PESI; TnI: serum Troponin I.

## Data Availability

Not applicable.

## References

[1] Demelo-Rodriguez P., Galeano-Valle F., Salzano A., Biskup E., Vriz O., Cittadini A., Falsetti L., Ranieri B., Russo V., Stanziola A.A. (2020). Pulmonary Embolism. Heart Fail. Clin..

[2] Konstantinides S.V., Meyer G., Becattini C., Bueno H., Geersing G.-J., Harjola V.-P., Huisman M.V., Humbert M., Jennings C.S., Jiménez D. (2019). 2019 ESC Guidelines for the diagnosis and management of acute pulmonary embolism developed in collaboration with the European Respiratory Society (ERS): The Task Force for the diagnosis and management of acute pulmonary embolism of the European Society of. Eur. Heart J..

[3] Falsetti L., Marra A.M., Zaccone V., Sampaolesi M., Riccomi F., Giovenali L., Guerrieri E., Viticchi G., D’Agostino A., Gentili T. (2022). Echocardiographic predictors of mortality in intermediate-risk pulmonary embolism. Intern. Emerg. Med..

[4] Casazza F., Becattini C., Bongarzoni A., Cuccia C., Roncon L., Favretto G., Zonzin P., Pignataro L., Agnelli G. (2012). Clinical features and short term outcomes of patients with acute pulmonary embolism. The Italian Pulmonary Embolism Registry (IPER). Thromb. Res..

[5] Piazza G. (2022). Off the beaten path: The need for innovation in medical therapy to improve outcomes in acute pulmonary embolism. Eur. Heart J. Acute Cardiovasc. Care.

[6] Gupta A., Day J.R., Streiff M.B., Takemoto C., Jung K., Abro C., Gehrie E., Bloch E., Tobian A., Goel R. (2020). Mortality and Associated Comorbidities Among Patients Hospitalized for Deep Vein Thrombosis and Pulmonary Embolism in the United States: Results from a Nationally Representative Database. Blood.

[7] Klok F.A., Hösel V., Clemens A., Yollo W.D., Tilke C., Schulman S., Lankeit M., Konstantinides S.V. (2016). Prediction of bleeding events in patients with venous thromboembolism on stable anticoagulation treatment. Eur. Respir. J..

[8] Chopard R., Bertoletti L., Piazza G., Jimenez D., Barillari G., Llamas P., Rubio C.M., Aujayeb A., Monreal M., Meneveau N. (2024). External validation of the PE-SARD risk score for predicting early bleeding in acute pulmonary embolism in the RIETE Registry. Thromb. Res..

[9] Jara-Palomares L., Jiménez D., Bikdeli B., Muriel A., Rali P., Yamashita Y., Morimoto T., Kimura T., Le Mao R., Riera-Mestre A. (2020). Derivation and validation of a clinical prediction rule for thrombolysis-associated major bleeding in patients with acute pulmonary embolism: The BACS score. Eur. Respir. J..

[10] Ruíz-Giménez N., Suárez C., González R., Nieto J., Todolí J., Samperiz Á., Monreal M. (2008). Predictive variables for major bleeding events in patients presenting with documented acute venous thromboembolism. Findings from the RIETE Registry. Thromb. Haemost..

[11] Milioglou I., Farmakis I., Wazirali M., Ajluni S., Khawaja T., Chatuverdi A., Giannakoulas G., Shishehbor M., Li J. (2022). Percutaneous thrombectomy in patients with intermediate- and high-risk pulmonary embolism and contraindications to thrombolytics: A systematic review and meta-analysis. J. Thromb. Thrombolysis.

[12] Lauder L., Pérez Navarro P., Götzinger F., Ewen S., Al Ghorani H., Haring B., Lepper P.M., Kulenthiran S., Böhm M., Link A. (2023). Mechanical thrombectomy in intermediate- and high-risk acute pulmonary embolism: Hemodynamic outcomes at three months. Respir. Res..

[13] Piazza G., Hohlfelder B., Jaff M.R., Ouriel K., Engelhardt T.C., Sterling K.M., Jones N.J., Gurley J.C., Bhatheja R., Kennedy R.J. (2015). A Prospective, Single-Arm, Multicenter Trial of Ultrasound-Facilitated, Catheter-Directed, Low-Dose Fibrinolysis for Acute Massive and Submassive Pulmonary Embolism. JACC Cardiovasc. Interv..

[14] Meyer G., Vicaut E., Danays T., Agnelli G., Becattini C., Beyer-Westendorf J., Bluhmki E., Bouvaist H., Brenner B., Couturaud F. (2014). Fibrinolysis for patients with intermediate-risk pulmonary embolism. N. Engl. J. Med..

[15] Jaff M.R., McMurtry M.S., Archer S.L., Cushman M., Goldenberg N., Goldhaber S.Z., Jenkins J.S., Kline J.A., Michaels A.D., Thistlethwaite P. (2011). Management of Massive and Submassive Pulmonary Embolism, Iliofemoral Deep Vein Thrombosis, and Chronic Thromboembolic Pulmonary Hypertension. Circulation.

[16] Giri J., Sista A.K., Weinberg I., Kearon C., Kumbhani D.J., Desai N.D., Piazza G., Gladwin M.T., Chatterjee S., Kobayashi T. (2019). Interventional Therapies for Acute Pulmonary Embolism: Current Status and Principles for the Development of Novel Evidence: A Scientific Statement From the American Heart Association. Circulation.

[17] Toma C., Bunte M.C., Cho K.H., Jaber W.A., Chambers J., Stegman B., Gondi S., Leung D.A., Savin M., Khandhar S. (2022). Percutaneous mechanical thrombectomy in a real-world pulmonary embolism population: Interim results of the FLASH registry. Catheter. Cardiovasc. Interv..

[18] Tu T., Toma C., Tapson V.F., Adams C., Jaber W.A., Silver M., Khandhar S., Amin R., Weinberg M., Engelhardt T. (2019). A Prospective, Single-Arm, Multicenter Trial of Catheter-Directed Mechanical Thrombectomy for Intermediate-Risk Acute Pulmonary Embolism. JACC Cardiovasc. Interv..

[19] Sista A.K., Horowitz J.M., Tapson V.F., Rosenberg M., Elder M.D., Schiro B.J., Dohad S., Amoroso N.E., Dexter D.J., Loh C.T. (2021). Indigo Aspiration System for Treatment of Pulmonary Embolism. JACC Cardiovasc. Interv..

[20] Dumantepe M., Teymen B., Akturk U., Seren M. (2015). The Efficacy of Rotational Thrombectomy on the Mortality of Patients with Massive and Submassive Pulmonary Embolism. J. Card. Surg..

[21] Meneveau N., Guillon B., Planquette B., Piton G., Kimmoun A., Gaide-Chevronnay L., Aissaoui N., Neuschwander A., Zogheib E., Dupont H. (2018). Outcomes after extracorporeal membrane oxygenation for the treatment of high-risk pulmonary embolism: A multicentre series of 52 cases. Eur. Heart J..

[22] Goldberg J.B., Giri J., Kobayashi T., Ruel M., Mittnacht A.J.C., Rivera-Lebron B., DeAnda A., Moriarty J.M., MacGillivray T.E. (2023). Surgical Management and Mechanical Circulatory Support in High-Risk Pulmonary Embolisms: Historical Context, Current Status, and Future Directions: A Scientific Statement From the American Heart Association. Circulation.

[23] Zuin M., Rigatelli G., Daggubati R., Nguyen T., Roncon L. (2020). Impella RP in hemodynamically unstable patients with acute pulmonary embolism. J. Artif. Organs.

[24] Patel M., Mujer M., John A., Darki A. (2022). VA-ECMO-assisted aspiration thrombectomy in a patient presenting with acute massive PE with absolute contraindications to thrombolytics. Catheter. Cardiovasc. Interv..

[25] Lashin H., Spiritoso R. (2019). Impella RP as Rescue Measure for Pulmonary Embolism With Hemodynamic Compromise. JACC Case Rep..

[26] Horowitz J.M., Jaber W.A., Stegman B., Rosenberg M., Fanola C., Bhat A.P., Gondi S., Castle J., Ahmed M., Brown M.A. (2024). Mechanical Thrombectomy for High-Risk Pulmonary Embolism: Insights From the US Cohort of the FLASH Registry. J. Soc. Cardiovasc. Angiogr. Interv..

[27] Gonsalves C.F., Gibson C.M., Stortecky S., Alvarez R.A., Beam D.M., Horowitz J.M., Silver M.J., Toma C., Rundback J.H., Rosenberg S.P. (2023). Randomized controlled trial of mechanical thrombectomy vs catheter-directed thrombolysis for acute hemodynamically stable pulmonary embolism: Rationale and design of the PEERLESS study. Am. Heart J..

[28] Saleh Velez F.G., Ortiz Garcia J.G. (2021). Management dilemmas in acute ischemic stroke and concomitant acute pulmonary embolism: Case series and literature review. eNeurologicalSci.

[29] Toma C. (2022). Impact of a blood return system on mechanical thrombectomy-associated blood loss and hemodynamic outcomes in a pulmonary embolism registry. Eur. Heart J..

[30] Monteleone P., Ahern R., Banerjee S., Desai K.R., Kadian-Dodov D., Webber E., Omidvar S., Troy P., Parikh S.A. (2024). Modern Treatment of Pulmonary Embolism (USCDT vs MT): Results From a Real-World, Big Data Analysis (REAL-PE). J. Soc. Cardiovasc. Angiogr. Interv..

[31] Abrahamian A., Khokher W., Ahmad R., Holtzapple Z., Patel R., Assaly R.A., Safi F. (2023). Catheter-directed thrombolysis vs. mechanical thrombectomy in treatment of acute pulmonary embolism: A systemic review and meta-analysis of dual arm studies. Chest.

[32] Fleitas Sosa D., Lehr A.L., Zhao H., Roth S., Lakhther V., Bashir R., Cohen G., Panaro J., Maldonado T.S., Horowitz J. (2022). Impact of pulmonary embolism response teams on acute pulmonary embolism: A systematic review and meta-analysis. Eur. Respir. Rev..

[33] Götzinger F., Lauder L., Sharp A.S.P., Lang I.M., Rosenkranz S., Konstantinides S., Edelman E.R., Böhm M., Jaber W., Mahfoud F. (2023). Interventional therapies for pulmonary embolism. Nat. Rev. Cardiol..

[34] Pruszczyk P., Klok F.K., Kucher N., Roik M., Meneveau N., Sharp A.S., Nielsen-Kudsk J.N.-K., Obradović S., Barco S., Giannini F. (2022). Percutaneous treatment options for acute pulmonary embolism: A clinical consensus statement by the ESC Working Group on Pulmonary Circulation and Right Ventricular Function and the European Association of Percutaneous Cardiovascular Interventions. EuroIntervention.

[35] Rajput F.A., Du L., Woods M., Jacobson K. (2020). Percutaneous Vacuum-Assisted Thrombectomy Using AngioVac Aspiration System. Cardiovasc. Revascularization Med..

[36] Pandya Y.K., Tzeng E. (2024). Mechanical thrombectomy devices for the management of pulmonary embolism. JVS-Vascular Insights.

[37] Srivathsa M., Illindala U., Al-Jadda A. (2023). A Differentiated Approach Delivering Targeted Thrombectomy for VTE. JACC Basic to Transl. Sci..

[38] Andersen A., Musialek P., Araszkiewicz A., Schultz J., Nielsen-Kudsk J.E., Tekieli L., Zajdel W., Sławek-Szmyt S., Taff Y., Weinberg I. (2023). First-in-Human Trial of Mechanical-Electric Thrombectomy in Acute Pulmonary Embolism. JACC Cardiovasc. Interv..

[39] Bashir R., Foster M., Iskander A., Darki A., Jaber W., Rali P.M., Lakhter V., Gandhi R., Klein A., Bhatheja R. (2022). Pharmacomechanical Catheter-Directed Thrombolysis With the Bashir Endovascular Catheter for Acute Pulmonary Embolism. JACC Cardiovasc. Interv..

[40] Sadeghipour P., Jenab Y., Moosavi J., Hosseini K., Mohebbi B., Hosseinsabet A., Chatterjee S., Pouraliakbar H., Shirani S., Shishehbor M.H. (2022). Catheter-Directed Thrombolysis vs Anticoagulation in Patients With Acute Intermediate-High–risk Pulmonary Embolism. JAMA Cardiol..

[41] Planer D., Yanko S., Matok I., Paltiel O., Zmiro R., Rotshild V., Amir O., Elbaz-Greener G., Raccah B.H. (2023). Catheter-directed thrombolysis compared with systemic thrombolysis and anticoagulation in patients with intermediate- or high-risk pulmonary embolism: Systematic review and network meta-analysis. Can. Med. Assoc. J..

[42] Kroupa J., Buk M., Weichet J., Malikova H., Bartova L., Linkova H., Ionita O., Kozel M., Motovska Z., Kocka V. (2022). A pilot randomised trial of catheter-directed thrombolysis or standard anticoagulation for patients with intermediate-high risk acute pulmonary embolism. EuroIntervention.

[43] Siordia J.A., Kaur A. (2022). Catheter-directed Thrombolysis versus Systemic Anticoagulation for Submassive Pulmonary Embolism: A Meta-Analysis. Curr. Cardiol. Rev..

[44] Blinc A., Francis C.W., Trudnowski J.L., Carstensen E.L. (1993). Characterization of ultrasound-potentiated fibrinolysis in vitro. Blood.

[45] Francis C.W., Blinc A., Lee S., Cox C. (1995). Ultrasound accelerates transport of recombinant tissue plasminogen activator into clots. Ultrasound Med. Biol..

[46] Kucher N., Boekstegers P., Müller O.J., Kupatt C., Beyer-Westendorf J., Heitzer T., Tebbe U., Horstkotte J., Müller R., Blessing E. (2014). Randomized, Controlled Trial of Ultrasound-Assisted Catheter-Directed Thrombolysis for Acute Intermediate-Risk Pulmonary Embolism. Circulation.

[47] Kuo W.T., Banerjee A., Kim P.S., DeMarco F.J., Levy J.R., Facchini F.R., Unver K., Bertini M.J., Sista A.K., Hall M.J. (2015). Pulmonary Embolism Response to Fragmentation, Embolectomy, and Catheter Thrombolysis (PERFECT). Chest.

[48] Avgerinos E.D., Jaber W., Lacomis J., Markel K., McDaniel M., Rivera-Lebron B.N., Ross C.B., Sechrist J., Toma C., Chaer R. (2021). Randomized Trial Comparing Standard Versus Ultrasound-Assisted Thrombolysis for Submassive Pulmonary Embolism. JACC Cardiovasc. Interv..

[49] Low Dose Catheter Directed Thrombolysis for Acute Pulmonary Embolism (BETULA). https://classic.clinicaltrials.gov/ct2/show/NCT03854266.

[50] Pulmonary Embolism—Thrombus Removal With Catheter-Directed Therapy (PE-TRACT). https://classic.clinicaltrials.gov/ct2/show/NCT05591118.

[51] Klok F.A., Piazza G., Sharp A.S.P., Ní Ainle F., Jaff M.R., Chauhan N., Patel B., Barco S., Goldhaber S.Z., Kucher N. (2022). Ultrasound-facilitated, catheter-directed thrombolysis vs anticoagulation alone for acute intermediate-high-risk pulmonary embolism: Rationale and design of the HI-PEITHO study. Am. Heart J..

[52] Low Dose Thrombolysis, Ultrasound Assisted Thrombolysis or Heparin for Intermediate High Risk Pulmonary Embolism (STRATIFY). https://clinicaltrials.gov/study/NCT04088292.

[53] Nguyen P.C., Stevens H., Peter K., McFadyen J.D. (2021). Submassive Pulmonary Embolism: Current Perspectives and Future Directions. J. Clin. Med..

[54] Tapson V.F., Sterling K., Jones N., Elder M., Tripathy U., Brower J., Maholic R.L., Ross C.B., Natarajan K., Fong P. (2018). A Randomized Trial of the Optimum Duration of Acoustic Pulse Thrombolysis Procedure in Acute Intermediate-Risk Pulmonary Embolism. JACC Cardiovasc. Interv..

[55] Pasha A.K., Siddiqui M.U., Siddiqui M.D., Ahmed A., Abdullah A., Riaz I., Murad M.H., Bjarnason H., Wysokinski W.E., McBane R.D. (2022). Catheter directed compared to systemically delivered thrombolysis for pulmonary embolism: A systematic review and meta-analysis. J. Thromb. Thrombolysis.

[56] Zuin M., Piazza G., Barco S., Bikdeli B., Hobohm L., Giannakoulas G., Konstantinides S. (2023). Time-based reperfusion in haemodynamically unstable pulmonary embolism patients: Does early reperfusion therapy improve survival?. Eur. Heart J. Acute Cardiovasc. Care.

[57] Patel I.J., Rahim S., Davidson J.C., Hanks S.E., Tam A.L., Walker T.G., Wilkins L.R., Sarode R., Weinberg I. (2019). Society of Interventional Radiology Consensus Guidelines for the Periprocedural Management of Thrombotic and Bleeding Risk in Patients Undergoing Percutaneous Image-Guided Interventions—Part II: Recommendations. J. Vasc. Interv. Radiol..

[58] Sanchez O., Charles-Nelson A., Ageno W., Barco S., Binder H., Chatellier G., Duerschmied D., Empen K., Ferreira M., Girard P. (2022). Reduced-Dose Intravenous Thrombolysis for Acute Intermediate–High-risk Pulmonary Embolism: Rationale and Design of the Pulmonary Embolism International THrOmbolysis (PEITHO)-3 trial. Thromb. Haemost..

[59] Weitz J.I., Chan N.C. (2020). Novel antithrombotic strategies for treatment of venous thromboembolism. Blood.

[60] Sevitt S. (1974). The structure and growth of valve-pocket thrombi in femoral veins. J. Clin. Pathol..

[61] Mackman N. (2012). New insights into the mechanisms of venous thrombosis. J. Clin. Investig..

[62] BRILL A., FUCHS T.A., SAVCHENKO A.S., THOMAS G.M., MARTINOD K., DE MEYER S.F., BHANDARI A.A., WAGNER D.D. (2012). Neutrophil extracellular traps promote deep vein thrombosis in mice. J. Thromb. Haemost..

[63] Kaplan M.J., Radic M. (2012). Neutrophil Extracellular Traps: Double-Edged Swords of Innate Immunity. J. Immunol..

[64] Fuchs T.A., Brill A., Duerschmied D., Schatzberg D., Monestier M., Myers D.D., Wrobleski S.K., Wakefield T.W., Hartwig J.H., Wagner D.D. (2010). Extracellular DNA traps promote thrombosis. Proc. Natl. Acad. Sci. USA.

[65] von Brühl M.-L., Stark K., Steinhart A., Chandraratne S., Konrad I., Lorenz M., Khandoga A., Tirniceriu A., Coletti R., Köllnberger M. (2012). Monocytes, neutrophils, and platelets cooperate to initiate and propagate venous thrombosis in mice in vivo. J. Exp. Med..

[66] Grover S.P., Olson T.M., Cooley B.C., Mackman N. (2020). Model-dependent contributions of FXII and FXI to venous thrombosis in mice. J. Thromb. Haemost..

[67] Sharman Moser S., Chodick G., Ni Y.G., Chalothorn D., Wang M.-D., Shuldiner A.R., Morton L., Salomon O., Jalbert J.J. (2022). The Association between Factor XI Deficiency and the Risk of Bleeding, Cardiovascular, and Venous Thromboembolic Events. Thromb. Haemost..

[68] Georgi B., Mielke J., Chaffin M., Khera A.V., Gelis L., Mundl H., van Giezen J.J.J., Ellinor P., Kathiresan S., Ziegelbauer K. (2019). Leveraging Human Genetics to Estimate Clinical Risk Reductions Achievable by Inhibiting Factor XI. Stroke.

[69] MORANGE P.E., TREGOUET D.A. (2011). Lessons from genome-wide association studies in venous thrombosis. J. Thromb. Haemost..

[70] Daghlas I., Gill D. (2023). Leveraging genetic predictors of factor XI levels to anticipate results from clinical trials. Eur. J. Neurol..

[71] Stavrou E., Schmaier A.H. (2010). Factor XII: What does it contribute to our understanding of the physiology and pathophysiology of hemostasis & thrombosis. Thromb. Res..

[72] Johnson C.Y., Tuite A., Morange P.E., Tregouet D.A., Gagnon F. (2011). The Factor XII −4C>T Variant and Risk of Common Thrombotic Disorders: A HuGE Review and Meta-Analysis of Evidence From Observational Studies. Am. J. Epidemiol..

[73] Willmann S., Marostica E., Snelder N., Solms A., Jensen M., Lobmeyer M., Lensing A.W.A., Bethune C., Morgan E., Yu R.Z. (2021). PK/PD modeling of FXI antisense oligonucleotides to bridge the dose-FXI activity relation from healthy volunteers to end-stage renal disease patients. CPT Pharmacomet. Syst. Pharmacol..

[74] Yu R.Z., Gunawan R., Post N., Zanardi T., Hall S., Burkey J., Kim T.-W., Graham M.J., Prakash T.P., Seth P.P. (2016). Disposition and Pharmacokinetics of a GalNAc3-Conjugated Antisense Oligonucleotide Targeting Human Lipoprotein (a) in Monkeys. Nucleic Acid Ther..

[75] van Es N., De Caterina R., Weitz J.I. (2023). Reversal agents for current and forthcoming direct oral anticoagulants. Eur. Heart J..

[76] Dilger A.K., Pabbisetty K.B., Corte J.R., De Lucca I., Fang T., Yang W., Pinto D.J.P., Wang Y., Zhu Y., Mathur A. (2022). Discovery of Milvexian, a High-Affinity, Orally Bioavailable Inhibitor of Factor XIa in Clinical Studies for Antithrombotic Therapy. J. Med. Chem..

[77] Wong P.C., Crain E.J., Bozarth J.M., Wu Y., Dilger A.K., Wexler R.R., Ewing W.R., Gordon D., Luettgen J.M. (2022). Milvexian, an orally bioavailable, small-molecule, reversible, direct inhibitor of factor XIa: In vitro studies and in vivo evaluation in experimental thrombosis in rabbits. J. Thromb. Haemost..

[78] Chan N.C., Weitz J.I. (2023). New Therapeutic Targets for the Prevention and Treatment of Venous Thromboembolism With a Focus on Factor XI Inhibitors. Arterioscler. Thromb. Vasc. Biol..

[79] A Study Comparing Abelacimab to Apixaban in the Treatment of Cancer-Associated VTE (ASTER). https://clinicaltrials.gov/study/NCT05171049.

[80] A Study Comparing Abelacimab to Dalteparin in the Treatment of Gastrointestinal/Genitourinary Cancer and Associated VTE (MAGNOLIA). https://www.clinicaltrials.gov/study/NCT05171075.

[81] Galli M., Laborante R., Ortega-Paz L., Franchi F., Rollini F., D’Amario D., Capodanno D., Tremoli E., Gibson C.M., Mehran R. (2023). Factor XI Inhibitors in Early Clinical Trials: A Meta-analysis. Thromb. Haemost..

[82] Nopp S., Kraemmer D., Ay C. (2022). Factor XI Inhibitors for Prevention and Treatment of Venous Thromboembolism: A Review on the Rationale and Update on Current Evidence. Front. Cardiovasc. Med..

[83] Ismayl M., Ismayl A., Hamadi D., Aboeata A., Goldsweig A.M. (2024). Catheter-directed thrombolysis versus thrombectomy for submassive and massive pulmonary embolism: A systematic review and meta-analysis. Cardiovasc. Revascularization Med..

[84] Silver M.J., Gibson C.M., Giri J., Khandhar S., Jaber W., Toma C., Mina B., Bowers T., Greenspon L., Kado H. (2023). Outcomes in High-Risk Pulmonary Embolism Patients Undergoing FlowTriever Mechanical Thrombectomy or Other Contemporary Therapies: Results From the FLAME Study. Circ. Cardiovasc. Interv..

[85] Ladenvall C., Gils A., Jood K., Blomstrand C., Declerck P.J., Jern C. (2007). Thrombin Activatable Fibrinolysis Inhibitor Activation Peptide Shows Association With All Major Subtypes of Ischemic Stroke and With TAFI Gene Variation. Arterioscler. Thromb. Vasc. Biol..

[86] Foley J.H., Kim P., Nesheim M.E. (2008). Thrombin-activable Fibrinolysis Inhibitor Zymogen Does Not Play a Significant Role in the Attenuation of Fibrinolysis. J. Biol. Chem..

[87] LEEBEEK F.W.G., VAN GOOR M.P.J., GUIMARAES A.H.C., BROUWERS G.J., DE MAAT M.P.M., DIPPEL D.W.J., RIJKEN D.C. (2005). High functional levels of thrombin-activatable fibrinolysis inhibitor are associated with an increased risk of first ischemic stroke. J. Thromb. Haemost..

[88] Leung L.L.K., Nishimura T., Myles T. (2008). Regulation of tissue inflammation by thrombin-activatable carboxypeptidase B (or TAFI). Adv. Exp. Med. Biol..

[89] Margetic S. (2012). Inflammation and haemostasis. Biochem. Medica.

[90] Leurs J., Hendriks D. (2005). Carboxypeptidase U (TAFIa): A metallocarboxypeptidase with a distinct role in haemostasis and a possible risk factor for thrombotic disease. Thromb. Haemost..

[91] Claesen K., Mertens J.C., Leenaerts D., Hendriks D. (2021). Carboxypeptidase U (CPU, TAFIa, CPB2) in Thromboembolic Disease: What Do We Know Three Decades after Its Discovery?. Int. J. Mol. Sci..

[92] Zhou J., Kochan J., Yin O., Warren V., Zamora C., Atiee G., Pav J., Orihashi Y., Vashi V., Dishy V. (2017). A first-in-human study of DS-1040, an inhibitor of the activated form of thrombin-activatable fibrinolysis inhibitor, in healthy subjects. J. Thromb. Haemost..

[93] Sakai N., Takeuchi M., Imamura H., Shimamura N., Yoshimura S., Naito H., Kimura N., Masuo O., Hirotsune N., Morita K. (2022). Safety, Pharmacokinetics and Pharmacodynamics of DS-1040, in Combination with Thrombectomy, in Japanese Patients with Acute Ischemic Stroke. Clin. Drug Investig..

[94] Vanassche T., Rosovsky R.P., Moustafa F., Büller H.R., Segers A., Patel I., Shi M., Miyoshi N., Mani V., Fayad Z. (2023). Inhibition of thrombin-activatable fibrinolysis inhibitor via DS-1040 to accelerate clot lysis in patients with acute pulmonary embolism: A randomized phase 1b study. J. Thromb. Haemost..

[95] Schmidt C.Q., Schrezenmeier H., Kavanagh D. (2022). Complement and the prothrombotic state. Blood.

[96] Delvasto-Nuñez L., Jongerius I., Zeerleder S. (2021). It takes two to thrombosis: Hemolysis and complement. Blood Rev..

[97] Berentsen S., Hill A., Hill Q.A., Tvedt T.H.A., Michel M. (2019). Novel insights into the treatment of complement-mediated hemolytic anemias. Ther. Adv. Hematol..

[98] Yerigeri K., Kadatane S., Mongan K., Boyer O., Burke L.L., Sethi S.K., Licht C., Raina R. (2023). Atypical Hemolytic-Uremic Syndrome: Genetic Basis, Clinical Manifestations, and a Multidisciplinary Approach to Management. J. Multidiscip. Healthc..

[99] Camous L., Veyradier A., Darmon M., Galicier L., Mariotte E., Canet E., Parquet N., Azoulay É. (2014). Macrovascular thrombosis in critically ill patients with thrombotic micro-angiopathies. Intern. Emerg. Med..

[100] Cervera R., Piette J., Font J., Khamashta M.A., Shoenfeld Y., Camps M.T., Jacobsen S., Lakos G., Tincani A., Kontopoulou-Griva I. (2002). Antiphospholipid syndrome: Clinical and immunologic manifestations and patterns of disease expression in a cohort of 1,000 patients. Arthritis Rheum..

[101] Ponce A., Rodríguez-Pintó I., Espinosa G., Quintas H., Erkan D., Shoenfeld Y., Cervera R. (2023). Pulmonary involvement in catastrophic antiphospholipid syndrome: A descriptive analysis from the “CAPS Registry”. Semin. Arthritis Rheum..

[102] Hussain H., Tarantino M.D., Chaturvedi S., McCrae K.R., Roberts J.C. (2022). Eculizumab for refractory thrombosis in antiphospholipid syndrome. Blood Adv..

[103] Santoro L., Falsetti L., Zaccone V., Nesci A., Tosato M., Giupponi B., Savastano M.C., Moroncini G., Gasbarrini A., Landi F. (2022). Impaired Endothelial Function in Convalescent Phase of COVID-19: A 3 Month Follow Up Observational Prospective Study. J. Clin. Med..

[104] Jayne D. (2019). Complement inhibition in ANCA vasculitis. Néphrologie Thérapeutique.

[105] Zelek W.M., Harrison R.A. (2023). Complement and COVID-19: Three years on, what we know, what we don’t know, and what we ought to know. Immunobiology.

[106] Høiland I.I., Liang R.A., Brækkan S.K., Pettersen K., Ludviksen J.K., Latysheva N., Snir O., Ueland T., Hindberg K., Mollnes T.E. (2019). Complement activation assessed by the plasma terminal complement complex and future risk of venous thromboembolism. J. Thromb. Haemost..

[107] Capecchi M., Ciavarella A., Artoni A., Abbattista M., Martinelli I. (2021). Thrombotic Complications in Patients with Immune-Mediated Hemolysis. J. Clin. Med..

[108] Ketenciler S., Gemalmaz H., Yücel C., Kayalar N. (2021). Successful treatment of massive pulmonary embolism in a pregnant woman complicated with atypical hemolytic uremic syndrome. J. Card. Surg..

[109] Bataillard A., Hebrard A., Gaide-Chevronnay L., Casez M., Dessertaine G., Durand M., Chavanon O., Albaladejo P. (2016). Extracorporeal life support for massive pulmonary embolism during pregnancy. Perfusion.

[110] Pfeffer M.A., Samuelson Bannow B.T. (2023). Pulmonary Embolism and Thrombocytopenia. PERT Consortium Handbook of Pulmonary Embolism.

[111] Hillmen P., Muus P., Dührsen U., Risitano A.M., Schubert J., Luzzatto L., Schrezenmeier H., Szer J., Brodsky R.A., Hill A. (2007). Effect of the complement inhibitor eculizumab on thromboembolism in patients with paroxysmal nocturnal hemoglobinuria. Blood.

[112] Loschi M., Porcher R., Barraco F., Terriou L., Mohty M., de Guibert S., Mahe B., Lemal R., Dumas P., Etienne G. (2016). Impact of eculizumab treatment on paroxysmal nocturnal hemoglobinuria: A treatment versus no-treatment study. Am. J. Hematol..

[113] Cançado R.D., Araújo A.d.S., Sandes A.F., Arrais C., Lobo C.L.D.C., Figueiredo M.S., Gualandro S.F.M., Saad S.T.O., Costa F.F. (2021). Consensus statement for diagnosis and treatment of paroxysmal nocturnal haemoglobinuria. Hematol. Transfus. Cell Ther..

[114] Araten D.J., Notaro R., Thaler H.T., Kernan N., Boulad F., Castro-Malaspina H., Small T., Scaradavou A., Magnan H., Prockop S. (2012). Thrombolytic therapy is effective in paroxysmal nocturnal hemoglobinuria: A series of nine patients and a review of the literature. Haematologica.

[115] Röth A., Bommer M., Hüttmann A., Herich-Terhürne D., Kuklik N., Rekowski J., Lenz V., Schrezenmeier H., Dührsen U. (2018). Eculizumab in cold agglutinin disease (DECADE): An open-label, prospective, bicentric, nonrandomized phase 2 trial. Blood Adv..

[116] Cofiell R., Kukreja A., Bedard K., Yan Y., Mickle A.P., Ogawa M., Bedrosian C.L., Faas S.J. (2015). Eculizumab reduces complement activation, inflammation, endothelial damage, thrombosis, and renal injury markers in aHUS. Blood.

[117] Noris M., Remuzzi G. (2014). Cardiovascular complications in atypical haemolytic uraemic syndrome. Nat. Rev. Nephrol..

[118] Meroni P.L., Macor P., Durigutto P., De Maso L., Gerosa M., Ferraresso M., Borghi M.O., Mollnes T.E., Tedesco F. (2016). Complement activation in antiphospholipid syndrome and its inhibition to prevent rethrombosis after arterial surgery. Blood.

[119] Kronbichler A., Frank R., Kirschfink M., Szilágyi Á., Csuka D., Prohászka Z., Schratzberger P., Lhotta K., Mayer G. (2014). Efficacy of Eculizumab in a Patient With Immunoadsorption-Dependent Catastrophic Antiphospholipid Syndrome. Medicine.

[120] López-Benjume B., Rodríguez-Pintó I., Amigo M.C., Erkan D., Shoenfeld Y., Cervera R., Espinosa G. (2022). Eculizumab use in catastrophic antiphospholipid syndrome (CAPS): Descriptive analysis from the “CAPS Registry”. Autoimmun. Rev..

[121] Siniscalchi C., Basaglia M., Riva M., Meschi M., Meschi T., Castaldo G., Di Micco P. (2023). Catastrophic Antiphospholipid Syndrome: A Review. Immuno.

